# Astrocyte heterogeneity across the brain and spinal cord occurs developmentally, in adulthood and in response to demyelination

**DOI:** 10.1371/journal.pone.0180697

**Published:** 2017-07-10

**Authors:** Hyesook Yoon, Grant Walters, Alex R. Paulsen, Isobel A. Scarisbrick

**Affiliations:** 1 Department of Physical Medicine and Rehabilitation, Rehabilitation Medicine Research Center, Mayo Clinic, Rochester, Minnesota, United States of America; 2 Department of Physiology and Biomedical Engineering, Mayo Clinic, Rochester, Minnesota, United States of America; 3 Neurobiology of Disease Program, Mayo Clinic, Rochester, Minnesota, United States of America; Medical University Vienna, Center for Brain Research, AUSTRIA

## Abstract

Astrocytes have emerged as essential regulators of function and response to injury in the brain and spinal cord, yet very little is known about regional differences that exist. Here we compare the expression of key astroglial markers (glial fibrillary acidic protein (GFAP) and Aldehyde Dehydrogenase-1 Family Member L1 (ALDH1L1)) across these disparate poles of the neuraxis, tracking their expression developmentally and in the context of demyelination. In addition, we document changes in the astrocyte regulatory cytokine interleukin 6 (IL-6), and its signaling partner signal transducer and activator of transcription 3 (STAT3), *in vivo and in vitro*. Results demonstrate that GFAP expression is higher in the developing and adult spinal cord relative to brain. Comparisons between GFAP and ALDH1L1 expression suggest elevations in spinal cord GFAP during the early postnatal period reflect an accelerated appearance of astrocytes, while elevations in adulthood reflect higher expression by individual astrocytes. Notably, increases in spinal cord compared to whole brain GFAP were paralleled by higher levels of IL-6 and STAT3. Equivalent elevations in GFAP, GFAP/ALDH1L1 ratios, and in IL-6, were observed in primary astrocyte cultures derived from spinal cord compared to cortex. Also, higher levels of GFAP were observed in the spinal cord compared to the brain after focal demyelinating injury. Altogether, these studies point to key differences in astrocyte abundance and the expression of GFAP and IL-6 across the brain and spinal cord that are positioned to influence regional specialization developmentally and responses occurring in the context of injury and disease.

## Introduction

As astrocytes emerge as key regulators of essential physiological functions in the CNS and its response to injury and disease, there is a growing need to more completely understand regional heterogeneity [1, 2]. Given the considerable cytoarchitectural and functional specialization that exists across the CNS, it is not surprising that complementary heterogeneity exists among astrocytes. Astrocyte heterogeneity includes inter- and intra-regional differences that relate to morphology [3, 4], developmental origin [5], gene expression profiles [6–10], cell-cell interactions [11], and responses to injury [12]. Distinctions in regional astrocyte properties also extend to the level of receptor and ion channel expression [13–16], transporter expression [17], gap junction connectivity [18], and calcium signaling properties [19–21]. Collectively these differences have important implications for not only the physiological properties of astrocytes, but also the contributions they make to CNS function, its development and plasticity, including responses to injury and disease [1].

The pleiotropic actions of astrocytes include participation in the maintenance of the blood brain barrier (BBB), regulation of blood flow, provision of trophic support, energy metabolism, antioxidant support, ion and water homeostasis, immune defense, synaptogenesis, synaptic transmission, and adult neurogenesis [22]. How astrocyte regional heterogeneity affects these functions across the CNS is an area of intense investigation. We are particularly interested in the fundamental differences that are likely to exist between astrocytes in the brain compared to the spinal cord, given the unique development and clinical conditions that can affect each region. Already, some differences between brain and spinal cord astrocytes have been described. For example, by taking up glutamate via glutamate transporter-1 (GLT-1 (EAAT2)), and glutamate-aspartate transporter (GLAST), astrocytes play a key role in maintaining CNS glutamate concentrations and protecting from excitotoxicity. Of particular interest in this regard, GLT-1 is responsible for more than 90% of glutamate uptake in the CNS and is expressed at 10-fold lower levels in the spinal cord compared to brain [17]. Heterogeneity in astrocyte lineage has also been established across the brain [23–25] and the spinal cord [2, 26–30].

Although some essential differences have been identified between brain and spinal cord astrocytes, there remains a relative paucity of information regarding the extent of their distinctive properties. Given the diverse roles of astrocytes in CNS development and disease a deeper appreciation of key differences may provide unique insights into developmental, injury and disease-related mechanisms, thereby informing directed therapies. To increase our understanding of brain relative to spinal cord astrocytes, we specifically quantified the expression of well studied astrocyte markers and signaling molecules, in the intact brain and spinal cord developmentally, in purified cultures of cortical or spinal cord astrocytes, and in the context of a focal demyelinating injury. The results of these studies point to significantly higher levels of GFAP, IL-6 and STAT3 in spinal cord compared to brain astrocytes developmentally and in adulthood. Moreover, spinal cord astrocytes express higher levels of GFAP in the context of a demyelinating injury. This new information will help guide efforts to target astrocytes in a temporally and regionally specific manner to foster neural development, plasticity and repair.

## Materials and methods

### Animal care and use

All mice used in these studies were C57BL6/J (Stock No. 000664, https://www.jax.org/strain/000664) obtained from Jackson Laboratory (Bar Harbor, ME). An equal number of male and female mice were studied to determine the appearance of astrocyte markers in the brain and spinal cord developmentally and in cell culture. Any differences in the response of astrocytes in the brain or spinal cord to a focal demyelinating injury focused on male mice. All mice were bred in our animal colony to obtain mice at each age examined, that is postnatal day 0 (P0), P7, P21 or P45, with P0 being considered the day of birth. All animal experiments were carried out with adherence to NIH Guidelines for animal care and safety and were approved by the Mayo Clinic Institutional Animal Care and Use Committee.

### Localization of GFAP and ALDH1L1 in the developing brain and spinal cord

To determine potential differences in the appearance of GFAP between the brain and spinal cord developmentally, 6 μm paraffin sections through the cervical spinal cord, or the rostral corpus callosum, were immunochemically stained for GFAP (GFAP, Z0334 Dako, Carpenteria, CA). All tissues were obtained from 4% paraformaldehyde fixed specimens. Since GFAP does not stain all astrocytes we stained a parallel set of tissues sections with an antibody recognizing aldehyde dehydrogenase 1 family, member L1 (Aldh1L1, ab87117, Abcam, Cambridge, MA). ALDH1L1 is a pan-astrocyte marker [31–33]. The abundance of staining in 3 to 4 animals per time point was quantified by determining the relative optical density (ROD) of antigen staining using KS-400 image analysis software (Carl Zeiss Vision, Hallbermoss, Germany) [34–36]. All ROD measurements were expressed as a percent of the area measured in each case.

### Quantification of GFAP, ALDH1L1, IL-6 and STAT3 in brain and spinal cord

#### Western blot

Western blots were used to quantify GFAP, ALDH1L1 and STAT3 protein levels. Whole brain or spinal cords were harvested from at least three individual mice on postnatal day P0, 7, 21 or 45 (adulthood). Brain and spinal cords at each time point were collectively homogenized in radio-immunoprecipitation assay buffer and 30 μg of protein resolved on sodium dodecyl sulfate-polyacrylamide gels (Bio-Rad Laboratories, Hercules, CA). Electroblotted membranes were used to sequentially probed for antigens of interest, including GFAP (ab7260, Abcam, Cambridge, MA), ALDH1L1 (ab87117, Abcam, Cambridge, MA), and the phosphorylated or total protein forms of STAT3 (sc-8059, sc-8019, Santa Cruz, Santa Cruz, CA). All Western blots were re-probed with an antibody recognizing β-actin (NB600-501, Novus Biologicals, Littleton, CO) to control for loading. Signal for each protein of interest in samples derived from mice were detected on the same film using species appropriate horseradish peroxidase-conjugated secondary antibodies (GE Healthcare, Buckinghamshier, UK) and chemiluminescent techniques (Pierce, Rockford, IL). Films were scanned and the ROD of bands in each case determined using Image Lab 2.0 software (Bio-Rad Laboratories). The ROD of each protein of interest was normalized to the ROD of actin detected on the same membrane and the mean and s.e. of these raw values across 3 to 4 independent Western blots used for analysis of the significance of the changes observed and for preparation of histograms.

#### RNA transcription

Real time RT-PCR was used to quantify any differences in the expression of GFAP, ALDH1L1, IL-6 or STAT3 between the brain and spinal cord developmentally or in adulthood. Whole brain or spinal cords were harvested from three individual mice on postnatal day P0, 7, 21 or 45 (adulthood) and RNA extracted with STAT-60 (Tel-Test, Friendswood, TX). The level of RNA encoding GFAP, ALDH1L1, IL-6 or STAT3 was determined in 0.10 μg of RNA in triplicate using an iCycler iQ5 system (BioRad) with primers described in Table 1 [35, 36]. The relative amount of RNA in each case was normalized to the constitutively expressed gene Rn18S. All PCR cycle conditions followed those recommended by BioRad using the iTaq Universal SYBR Green One-Step kit (#172–5151) for conventional primers, and the iTaq Universal Probes One-Step Kit (#172–5141) for hybridization probes.

**Table 1:** Primers used for quantitative real-time PCR.

Gene	Accession number	Primer Sequence Forward/Reverse
**ALDH1L1**	NM_027406.1	Applied Biosystems, Assay ID: Mm03048957_m1
**GFAP**	NM_010277.2	GCAGATGAAGCCACCCTGG / GAGGTCTGGCTTGGCCAC (IDT)
**IL-6**	NM_031168.1	Applied Biosystems, Assay ID: Mm00446190_m1
**Rn18S**	NR_003278.3	Applied Biosystems, Assay ID: Mm03928990_g1
**STAT3**	NM_001048139.1	Applied Biosystems, Assay ID: Mm01219775_m1

All PCR primers were obtained from Integrated DNA Technologies (IDT) unless otherwise indicated.

### Astroglial cultures

Primary brain or spinal cord astrocytes were purified from mixed glial cultures prepared from the cortices or whole spinal cord of P1 male and female mice as we have previously described [35–40]. In all cases, mixed glial cultures were grown in DMEM, containing 2 mM Glutamax, 1 mM sodium pyruvate, 20 mM HEPES, and 10% heat inactivated fetal calf serum (Atlanta Biologicals, Lawrenceville, GA). Purified astrocytes were obtained from 10 days *in vitro* mixed cultures by overnight shaking to remove oligodendrocyte progenitor cells and sequential panning on non-tissue culture treated plastic to eliminate microglia. Astrocytes were trypsinized and plated across poly-L-lysine (Sigma, St. Louis, MO) coated 6 well plates in the same media at a density of 4.7 x 10^4^ cells/cm^2^. Alternatively, purified astrocytes were plated on poly-L-lysine coated coverslips at a density of 1.6 x 10^4^ cells/cm^2^. Twenty-four h later, media was replaced with defined Neurobasal A media containing 1% N2, 2% B27, 50 U/mL penicillin/streptomycin, 2 mM Glutamax, 1 mM sodium pyruvate, 0.45% glucose, and 50 μM β-mercaptoethanol (Sigma Aldrich, USA). All cells were maintained at 37°C in 95% air and 5% CO_2_, and all cell culture experiments were repeated independently at least 3 times. Cultures were harvested into radio-immunoprecipitation assay buffer for quantification of GFAP, STAT3 or ALDH1L1 expression by Western blot as described above. In parallel experiments, cells were harvested into RNA STAT-60 for RNA isolation and quantification using real time RT-PCR. In all cases, cell culture supernatants were snap frozen and stored at -70 for quantification of IL-6 by Enzyme Linked Immunosorbant Assay (ELISA) (eBioscience, San Diego, CA). In other cases, coverslips containing either purified brain or spinal cord astrocytes were fixed with 2% paraformaldehyde and stained for GFAP or ALDH1L1 and counterstained with DAPI. To enumerate the number of cells positive for each antigen, five 20X fields encompassing the poles and center of each coverslip were captured digitally. The mean number of GFAP or ALDH1L1+ cells was also enumerated and expressed as a ratio of the number of DAPI cells present in each field. 98.5% of DAPI+ cells in each paradigm were also positive for the pan astrocyte marker ALDH1L1 [36].

### Lysolecithin induced focal demyelinating lesions

To determine any differences in astroglial responses occurring after focal demyelination in the brain or spinal cord we compared changes in GFAP and ALDH1L1 14d after induction of demyelination in the corpus callosum, or the dorsal column white matter of the spinal cord in 10–12 wk old male C57BL6/J mice. 2 μl of a 1% solution of lysophosphatidyl choline (L-4129, Sigma-Aldrich, St. Louis, MO) was injected into the T11-T12 dorsal column (n = 4), or into the corpus callosum (n = 5), using a 30 to 70 μm glass micropipette at a rate of 0.25 μl/min using a stereotaxic microinjection system (Stoelting, Inc., Wood Dale, IL). Corpus callosum injections were stereotaxically targeted using coordinates of 1 mm anterior to the Bregma, 1mm lateral, and 2.3 mm deep from the skull surface [41]. In all cases, mice were anesthetized with ketamine (1mg/kg, Fort Dodge Animal Health, Fort Dodge, IA) and xylazine (0.125 mg/kg, Akom, Inc., Decatur, IL). Buprenorphine (0.05 mg/kg, Hospira, Lake Forest, IL) was given intraperitoneally postoperatively to minimize discomfort. Following a 14 d period of recovery mice were perfused transcardially with 4% paraformaldehyde. For analysis of focal spinal cord lesions, a 2 mm block of spinal cord encompassing the site of lysolecithin injection was embedded in paraffin. For evaluation of focal corpus callosum lesions, the brain was bisected at the level of injection with both halves embedded in paraffin. In each case, 6 μm paraffin sections were stained for GFAP or ALDH1L1 with a nuclear counterstain and quantified as described above.

### Statistical comparisons

All data were expressed as mean ± s.e. Comparisons between multiple groups were made using a One-Way Analysis of Variance (ANOVA) and the Newman Keuls post-hoc test. When multiple comparison data was not normally distributed, the Kruskal-Wallis ANOVA on Ranks was applied with Dunn’s method. For pairwise comparisons between two groups two-tailed unpaired Student’s t-test was applied. Statistical significance was set at P < 0.05. In each Figure significant differences within a region relative to time P0 are indicated by asterisks, while differences at a given time point between brain and spinal cord are indicted by a line with asterisks.

## Results

### Dynamics of expression of astroglial markers in the developing brain and spinal cord

**Fig 1:**
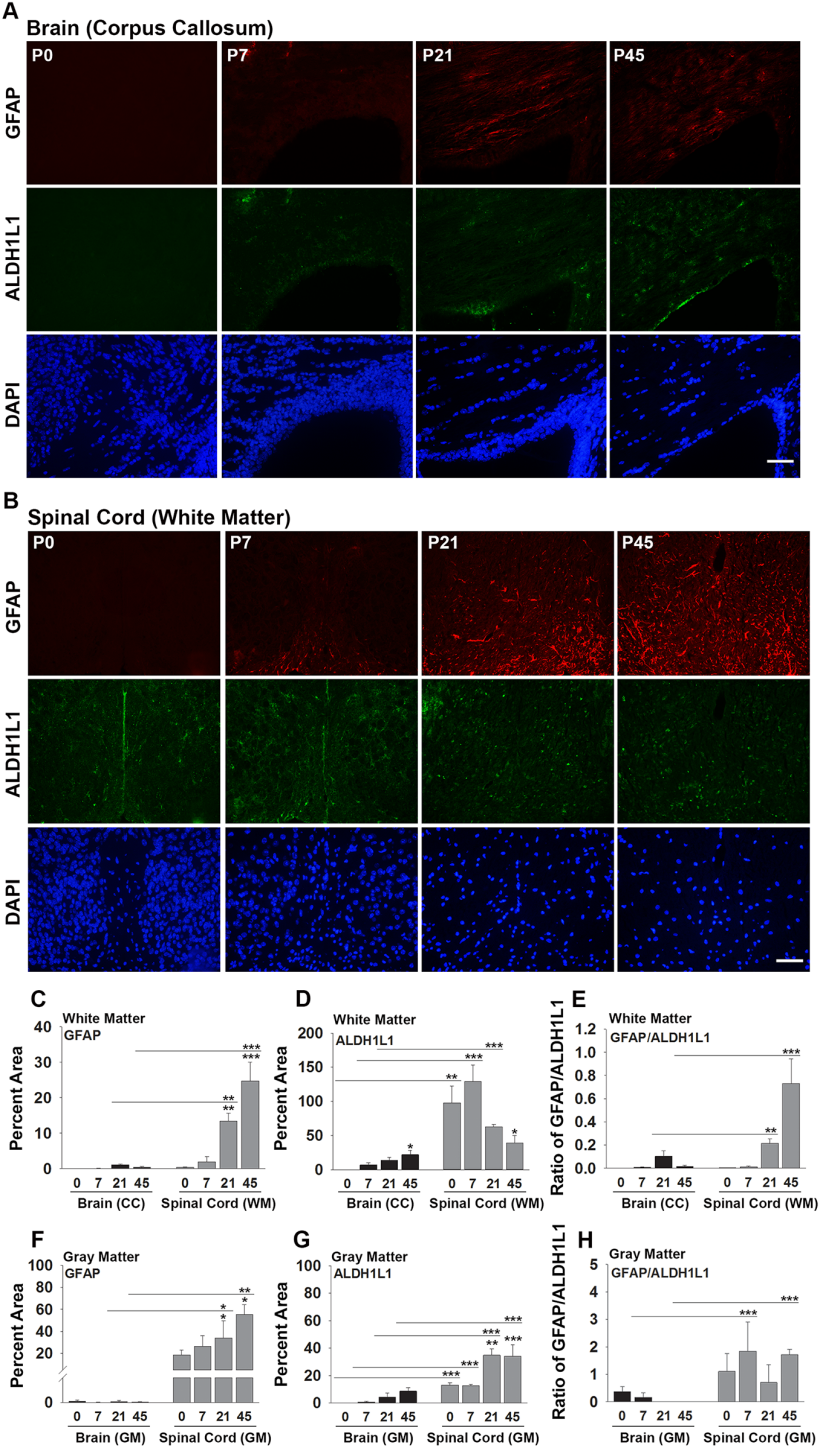
Astrocytes appear earlier in the developing spinal cord compared to brain and express higher levels of GFAP in adulthood. Photomicrographs show immunofluorescence for GFAP, ALDH1L1 and DAPI in (A) the corpus callosum of the brain, and (B) the dorsal column white matter (WM) of the spinal cord from P0 to adulthood (P45). Histograms (C and D) show the percent area stained for GFAP or ALDH1L1 in white matter of the brain (corpus callosum (CC), or in white matter of the spinal cord (dorsal column). Histograms (F and G) show the percent area stained for GFAP or ALDH1L1 in the gray matter (GM) of the brain (cingulate cortex) or gray matter of the spinal cord (ventral horn). (E) and (H) show the ratio of GFAP to ALDH1L1 immunoreactivity in each case. ALDH1L1-immunopositive astrocytes appear earlier in the spinal cord compared to brain and express higher levels of GFAP in adulthood. (*P < 0.05, **P ≤ 0.01, ***P ≤ 0.001, NK, scale bar = 50 μm).

To determine whether differences exist in the abundance of astrocytes in the white matter of the brain and spinal cord developmentally or in adulthood, we quantified immunoreactivity for GFAP, or the pan-astroglial marker ALDH1L1, in the corpus callosum or the spinal cord dorsal column white matter on P0, 7, 21 or 45 (adulthood) (Fig 1A to 1E). On P0, ALDH1L1+ cells were readily observed in the spinal cord white matter, but not in the corpus callosum. ALDH1L1-immunoreactivity was 97-fold more abundant in the spinal cord dorsal column compared to the corpus callosum on P0 (P = 0.001), 19-fold higher on P7 (P ≤ 0.001), and 4.6-fold higher on P21 (P ≤ 0.001, Student’s t-test). By P45, ALDH1L1 levels were similar in the corpus callosum and spinal cord white matter. GFAP-immunoreactivity was very low in the corpus callosum and in the spinal cord white matter at birth and on P7. By P21 however, GFAP levels were 13-fold higher in the spinal cord dorsal column white matter compared to the corpus callosum, and 67-fold higher in the adult, (P ≤ 0.005, Student’s t-test). Comparisons of GFAP/ALDH1L1 ratios were made to distinguish whether increases in GFAP observed in the spinal cord might be related to an increase in the number of astrocytes and/or could be related to an increase in GFAP expression per ALDH1L1 astrocyte. Since the GFAP/ALDH1L1 ratio was elevated by 44.5-fold in the spinal cord white matter compared to the corpus callosum on P45 (P < 0.001), higher levels of expression of GFAP per astrocyte in the adult spinal cord is suggested.

To determine if the increases in GFAP and GFAP/ALDH1L1 ratios that we observe in the spinal cord white matter compared to brain also occur in gray matter, measurements of GFAP and ALDH1L1 immunoreactivity were made in the cingulate cortex overlying the corpus callosum, and in the ventral horn gray matter of the spinal cord (Fig 1F to 1H). As was the case for white matter, levels of GFAP-immunoreactivity in spinal cord gray matter were also significantly higher compared to brain on P21 and P45 (P ≤ 0.04, Student’s t-test). Levels of ALDH1L1 were also significantly higher in the gray matter of the spinal cord compared to the cingulate cortex gray matter on P0, P7, P21 and in adulthood (P45) (P ≤ 0.001, Student’s t-test). Similar to observations across brain and spinal cord white matter, the ratio of GFAP to ALDH1L1 was also higher in the gray matter of the spinal cord compared to brain on P7 and P45 (P ≤ 0.04, Student’s t-test).

### Dynamics of expression of astroglial markers in the developing brain and spinal cord

**Fig 2:**
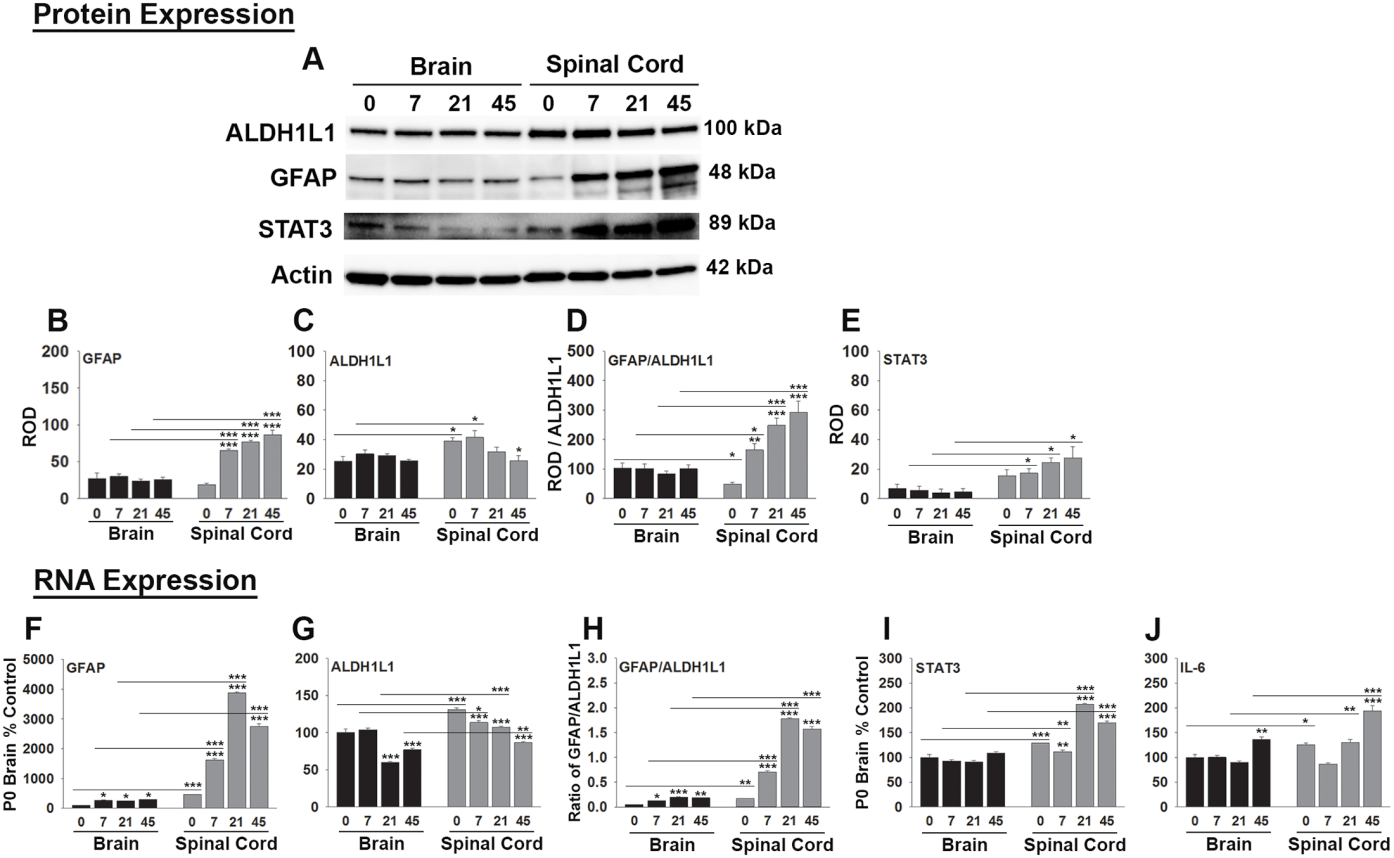
Astrocytes are more abundant in the spinal cord relative to brain in the early postnatal period and express higher levels of GFAP, STAT3 and IL-6 from P7 to adulthood. Western blots (A) and associated histograms (B to E) illustrate that (C) ALDH1L1 protein, a marker of astrocytes appears earlier developmentally in the spinal cord relative to brain with higher levels at birth and at P7. In addition, GFAP protein levels (B) and the ratio of GFAP/ALDH1L1 (D) are higher in the spinal cord from P7 through P45. (E) STAT3 levels are also elevated in the spinal cord relative to brain on P21 and P45. P-STAT3 was not detected in the brain or spinal cord under the conditions of this study (blot not shown). ROD readings in each case were normalized to actin on the same membrane to control for loading. (F) GFAP RNA transcription increased after P0 in both the brain and spinal cord, although increases in the spinal cord reached significantly higher levels compared to brain at all time points examined. (G) ALDH1L1 RNA was higher in the spinal cord compared to brain at P0 through P45. (H) The ratio of GFAP/ALDH1L1 was increased during the postnatal period in both brain and spinal cord although such increases were significantly higher in the spinal cord compared to brain at all postnatal ages. (I) STAT3 and (J) IL-6 RNA were higher in the spinal cord compared to brain at birth on P21 and in adulthood. (*P < 0.05, **P ≤ 0.01, ***P ≤ 0.001 NK).

Given the significantly higher levels of GFAP expression we observed in the spinal cord compared to brain white matter immunochemically, we evaluated whether similar differences would be detected in whole brain or spinal cord homogenates using quantitative Western blot, and real time PCR (Fig 2). The mean levels of GFAP protein in the brain and spinal cord were similar at P0. However, by P0 GFAP RNA expression was already 4.6-fold higher in spinal cord compared to brain (P < 0.001). Whereas any changes in the abundance of GFAP protein in brain homogenates were minor between birth through P45, in spinal cord GFAP protein levels rose sharply after birth, reaching approximately 3.5 to 4.7-fold higher levels between P7 and adulthood (P < 0.001, NK). GFAP RNA expression in the brain did increase by 2.4- to 2.9-fold between P0 and adulthood. Over the same period, increases in GFAP RNA expression in the spinal cord were much larger in magnitude reaching 3.5- to 8.5-fold (P < 0.001, NK), with levels reach being significantly higher than brain at every time point examined, including in the adult spinal cord by 9.6-fold (P < 0.001).

We used the pan-astrocyte marker ALDH1L1 to evaluate the appearance of brain and spinal cord astrocytes developmentally and to shed light on the potential abundance of GFAP expression by individual astrocytes (Fig 2). Spinal cord ALDH1L1 protein was already higher in whole spinal cord homogenates compared to brain at term and on P7 (~1.4-fold), but levels decreased to a level comparable to that in brain thereafter. ALDH1L1 RNA was higher in spinal cord compared to brain at these time points, as well as P21 and P45 (1.1–1.5-fold, P < 0.05) (Fig 2G). Ratios of GFAP to ALDH1L1 protein, suggest that GFAP expression per astrocyte is 2-fold higher in brain compared to spinal cord at birth, but thereafter spinal cord astrocytes express between 3- and 8-fold higher levels of GFAP by P7 through P45 compared to brain astrocytes (P < 0.05) (Fig 2D and 2H). GFAP/ALDH1L1 ratios suggest that the greatest increased in GFAP RNA per astrocyte occurs by P21 and in adulthood.

To gain insight into potential molecular differences contributing to higher levels of GFAP in the spinal cord relative to brain we examined expression of IL-6, a pro-inflammatory cytokine that is produced by astrocytes and can drive GFAP expression in an autocrine or paracrine fashion [35, 36]. IL-6 RNA expression was elevated in the spinal cord relative to brain by 1.2-fold at term, by 1.4-fold on P21, and by 1.4-fold in adulthood (P < 0.05) (Fig 2J).

**Fig 3:**
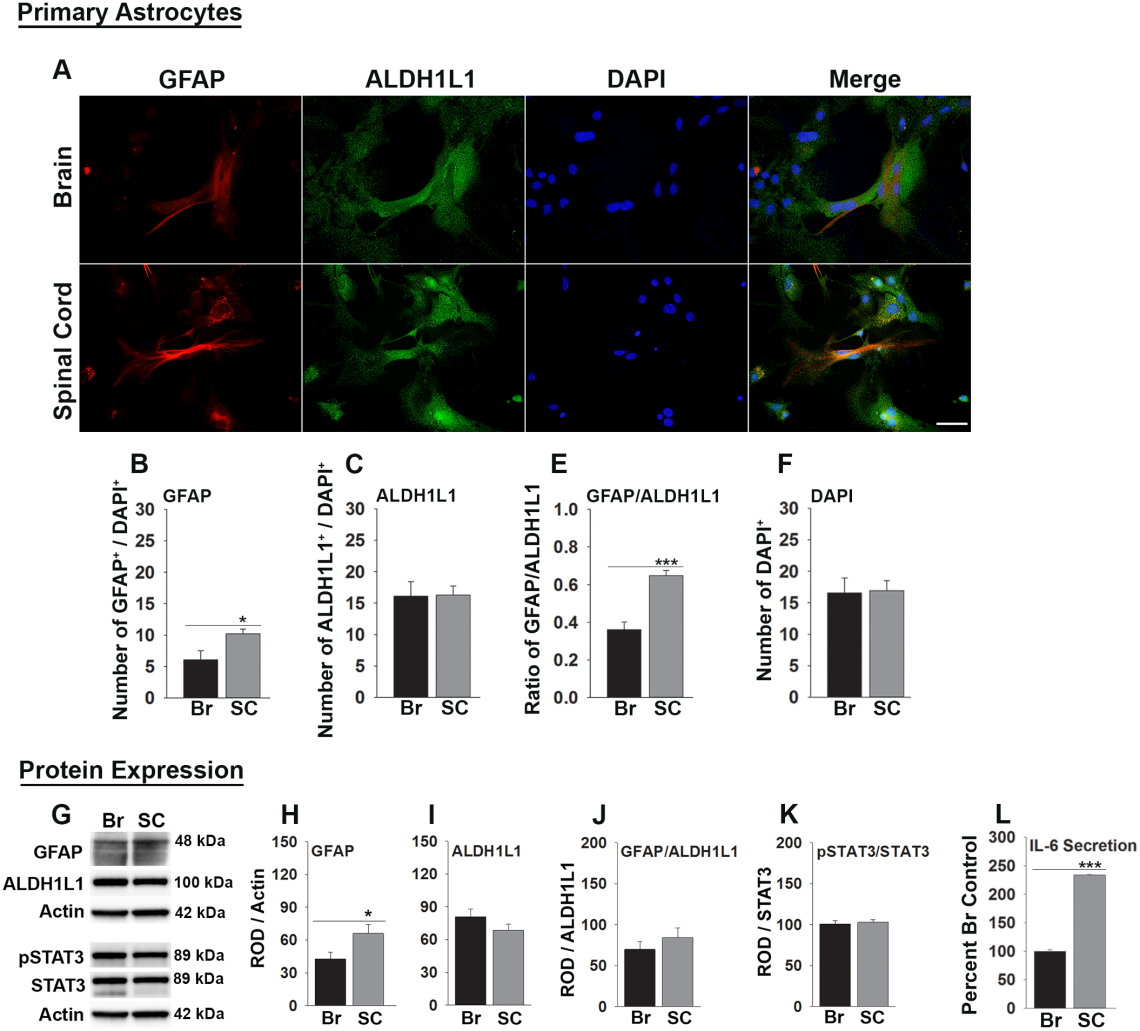
GFAP and IL-6 are elevated in cultures of spinal cord compared to cortical astrocytes. (A) Photomicrographs show GFAP and ALDH1L1 co-immunofluorescence in primary cultures of astrocytes purified from brain or spinal cord. (B-F) Histograms show counts of GFAP+ or ALDH1+ cells expressed as a ratio to the total number of DAPI+ cells in the same microscopic fields. The number of GFAP+ and GFAP/ALDH1L1+ cell was elevated in spinal cord astrocyte cultures compared to those derived from brain. (G, H) Analysis of protein homogenates from brain or spinal cord astrocyte cultures also demonstrated higher levels of GFAP expression, but no differences in ALDH1L1 (I), GFAP/ALDH1L1 ratios (J) or pSTAT3/STAT3 ratios. (L) IL-6 was higher in the supernatants of spinal cord compared to brain astrocyte cultures (P ≤ 0.001), while no differences in STAT3 were observed (K). (*P < 0.05, ***P ≤ 0.001, Students t-test). Scale bar = 50μm.

STAT3 is the canonical signaling partner for IL-6 and was also elevated at a protein and RNA level in whole spinal cord compared to brain homogenates on P0 through adulthood (Fig 3A, 3E and 3I). Elevations in spinal cord STAT3 relative to brain ranged from 2 to 6-fold with highest levels occurring by P21 and in adulthood. The phosphorylated form of STAT3 was not detectable in samples prepared from the intact brain and spinal cord at any stage of development examined (data not shown).

### Higher levels of GFAP and IL-6 are associated with spinal cord compared to brain-derived astrocytes

To begin to address whether the higher levels of GFAP in spinal cord compared to brain astrocytes may reflect environmental factors, or be inherent to the cells themselves, we evaluated expression patterns in purified astrocyte cultures derived from P0 mouse cortex or spinal cord (Fig 3). First, while counts of ALDH1L1+ or DAPI+ cells per μm^2^ were similar in 3 DIV cultures of brain and spinal cord astrocytes, those derived from the spinal cord had 40% more GFAP+ cells (P < 0.011). These differences were also reflected in 1.8-fold increases in the ratio of GFAP/ALDH1L1 in astrocyte cultures derived from the spinal cord compared to the brain (P ≤ 0.001). In parallel, higher levels of GFAP protein, but similar levels of ALDH1L1, were detected by Western blot in protein isolated from parallel spinal cord compared to brain astrocyte cultures (P < 0.048). IL-6 was also 2.3-fold higher in spinal cord astrocyte cell culture supernatants relative to that detected in cortical astrocyte supernatants (P ≤ 0.001). Levels of total or phosphorylated STAT3 did not differ between cultures of brain and spinal cord astrocytes.

### Toxin-mediated demyelination elicits more GFAP reactivity in spinal cord compared to the corpus callosum

**Fig 4:**
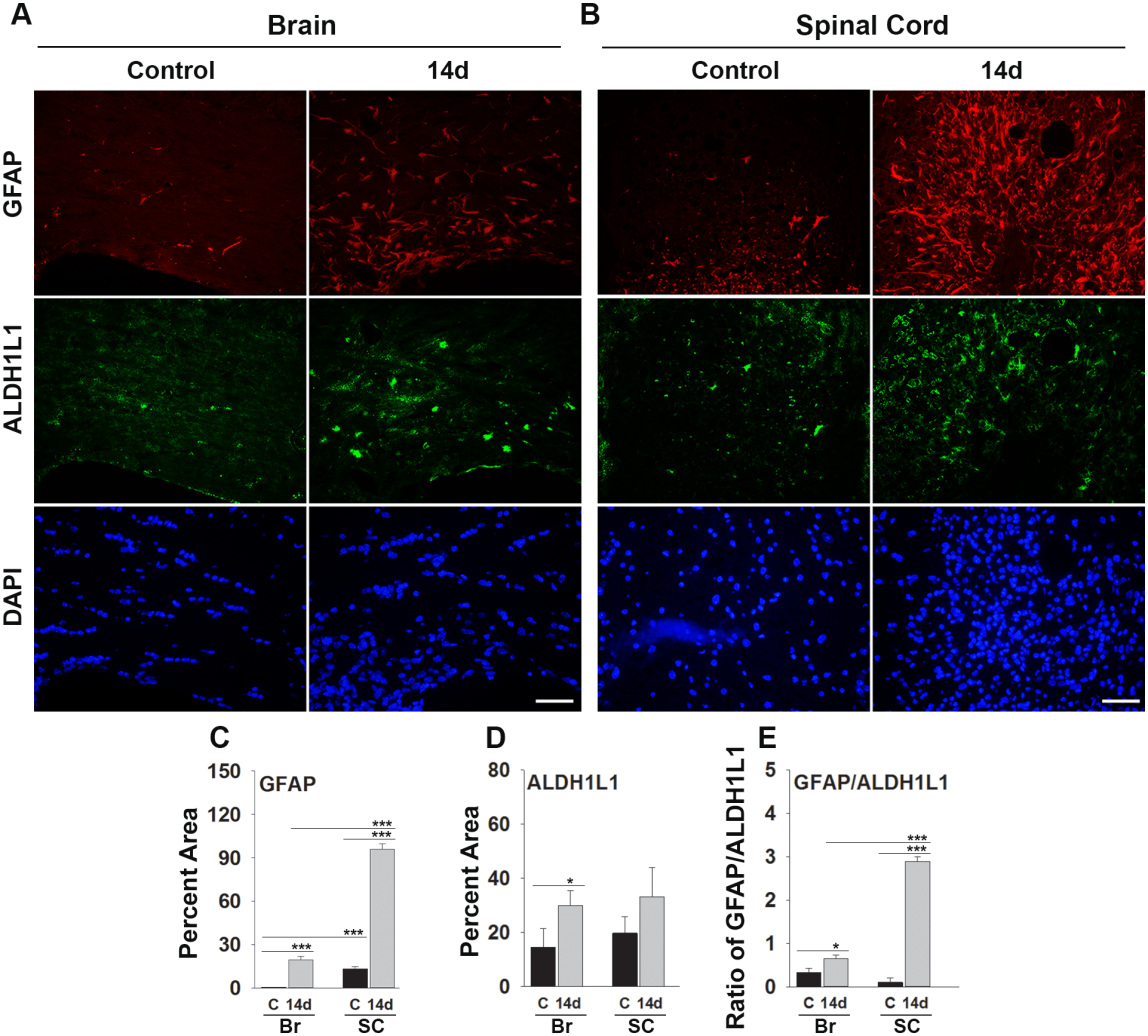
Increases in GFAP after demyelinating injury are greater in the spinal cord compared to brain. Photomicrographs show immunoreactivity for GFAP or ALDH1L1 in (A) the corpus callosum, or (B) the dorsal column white matter of adult mice at base line, and at 14 d after microinjection of the demyelinating agent lysolecithin. Histograms show the percent area of GFAP immunofluorescence, and expression of GFAP/ALDH1L1+ astrocyte, was significantly greater in the spinal cord compared to the corpus callosum 14d post-lysolecithin lesion. (*P < 0.05, **P ≤ 0.01, ***P ≤ 0.001, Students t-test), Scale bar = 50μm.

To determine whether the enrichment of GFAP observed in the adult spinal cord relative to brain would also be reflected in the response of each region to injury, we examined levels of GFAP and ALDH1L1 immunofluorescence 14d after induction of a focal demyelinating lesion using lysolecithin (Fig 4). As expected based on findings at P45, in the 12 wk old mice used for the lysolecithin studies we observed a 26-fold greater area of white matter encompassed by GFAP staining in the spinal cord relative to brain (P ≤ 1.8x10^-6^, Student’s t-test). Also, as anticipated, fourteen days after lysolecithin-mediated demyelination, increases in GFAP occurred in both the brain (39-fold) and spinal cord (7.4-fold) relative to baseline (P ≤ 0.02x10^-6^, Student’s t-test). ALDH1L1 immunofluorescence did not differ at baseline between brain and spinal cord but was elevated in both at 14d after lysolecithin-mediated demyelination, when nearly 2-fold increases were seen reaching statistical significance in the brain (P = 0.01, Student’s t-test). The dynamic changes observed in GFAP and ALDH1L1 between brain and spinal cord were reflected in significantly different GFAP/ALDH1L1 ratios. At 14 d after lysolecithin injection, the GFAP/ALDH1L1 ratio was increased by 2-fold in the corpus callosum and by 28-fold in the spinal cord. The higher fold change observed in GFAP/ALDH1L1 ratios in the spinal cord relative to the corpus callosum at 14d after lysolecithin injection suggests that spinal cord astrocytes express higher levels of GFAP in the context of a demyelinated lesion (P < 0.001).

## Discussion

The results of the current study demonstrate that astrocytes appear earlier and are more abundant in the developing spinal cord compared to the brain, but show similar abundance in adulthood. However, in adulthood spinal cord astrocytes express higher levels of GFAP compared to those in the brain. The increases in GFAP observed in the spinal cord were paralleled by increases in the GFAP regulatory cytokine, IL-6, and its signaling partner STAT3. Moreover, the levels of GFAP expression per astrocyte were also significantly higher in spinal cord compared to brain in response to focal demyelination. The elevations we observe in GFAP in the spinal cord compared to brain developmentally, in adulthood, and in response to injury highlight not only regional heterogeneity but also the need to better understand the physiological roles of this well studied, but poorly understood protein across the neuraxis.

### Astrocytes appear earlier in the developing spinal cord compared to brain

Astrocytes play key roles in CNS development ranging from synapse formation to myelination [42]. Comparisons of the abundance of the astrocyte marker ALDH1L1 between the brain and spinal cord developmentally point to the earlier appearance of spinal cord astrocytes. First, levels of ALDH1L1 immunoreactivity were higher in spinal cord white matter compared to the corpus callosum, with 97-fold higher levels at birth, 19-fold higher levels at P7, and 4.5-fold higher levels by P21. Near parallel elevations were observed in ALDH1L1 immunoreactivity in the spinal cord ventral horn gray matter compared to the cingulate cortex, with 216-fold higher levels at birth, 18-fold higher levels at P7, 8-fold higher levels at P21, and 4-fold higher levels in adulthood. Suggesting that increases in ALDH1L1 in the spinal cord compared to brain are not likely to be limited to specific regions, we observed corresponding increases in ALDH1L1 protein and RNA in homogenates of whole spinal cord compared to whole brain at P0 and P7. Given the broad roles of astrocytes in CNS development, these findings have significant implications for understanding development and regional specialization of the brain and spinal cord.

### Spinal cord astrocytes express higher levels of GFAP in adulthood compared to those from brain

The current studies uncovered several lines of evidence suggesting that astrocytes in the healthy spinal cord express higher levels of GFAP compared to those in brain. First, we observed substantially higher levels of GFAP in protein and RNA purified from spinal cord compared to brain starting on P7 through adulthood. Comparisons of GFAP to ALDH1L1 in the same samples suggest that elevations in GFAP in spinal cord relative to brain reflect higher levels of expression per astrocyte. These findings were corroborated by immune-localization of GFAP that showed 13-fold higher levels in spinal cord white matter compared to brain on P21, and 67-fold higher levels in adulthood. Immunoreactivity for GFAP was also elevated in the gray matter of the spinal cord relative to the cingulate cortex on P21 and in adulthood. Substantial increases in the ratio of GFAP/ALDH1L1 across the spinal cord gray and white matter developmentally and in adulthood, suggests that increases in spinal cord GFAP likely reflect greater expression per astrocyte, rather than simply increases in astrocyte abundance. Findings that GFAP levels per ALDH1L1 astrocyte are also higher in primary astrocyte cultures purified from the spinal cord compared to the brain, further supports the idea that spinal cord astrocytes express higher levels of GFAP *in vivo* and *in vitro*. The current studies also suggest the need for future efforts to determine how these observations extend to astrocytes derived from additional regions of the CNS.

The significance of higher levels of GFAP expression in the spinal cord compared to brain astrocytes from P21 through adulthood remains to be determined. GFAP is a type III intermediate filament protein and an important component of the astrocyte cytoskeleton that participates in the maintenance of mechanical strength and shape [43]. Therefore, higher GFAP levels per astrocyte in the spinal cord may relate to the role of GFAP in the maintenance of mechanical strength. Most certainly the spinal cord is subject to significantly more bending and torque than is the brain under everyday conditions for which GFAP-related enhancements in mechanical strength would be beneficial. In addition, there are several other functions attributed to GFAP based on careful analysis of mice either lacking GFAP [44–47], or with GFAP over expression [48, 49]. This includes roles for GFAP in several processes that would clearly differentiate the physiology of the spinal cord from the brain and its response to injury and disease including cell division, process extension, glutamate uptake, neurite outgrowth, signal transduction, synapse formation, myelination, reactive gliosis, and functioning of the blood brain barrier [50]. Since considerable inter-species differences exist in astrocyte form and function [51], the current findings in mice raise questions whether similar increases in GFAP occur in the human spinal cord relative to brain. Although GFAP is extensively used as a cell marker, the full scope of its actions remains poorly understood. The very clear differences observed in the level of GFAP relative to the astrocyte marker ALDH1L1 between spinal cord and brain, taken with many emerging lines of evidence regarding the roles of astrocytes in disease, underscores the importance of continuing to define the physiological properties of this protein, and the implications that its differential expression may have for CNS developmental specialization and regional vulnerabilities to injury and disease.

### Spinal cord astrocytes express higher levels of IL-6 and STAT3 compared to those derived from brain

IL-6 is a pleiotropic cytokine up regulated in reactive astrocytes [52]. IL-6 is also produced by perivascular astrocytes and can increase permeability of the BBB [53]. IL-6 is established to play a key role in astrogliosis and recovery of function in the context of spinal cord injury (SCI). For example, blockade of IL-6 after SCI reduces astrogliosis, inflammation and connective tissue scarring, and improves behavioral outcomes [54–56]. Given the ability of IL-6 to directly up regulate GFAP [36], and its link to SCI and repair, we compared the expression of IL-6 in the developing and adult spinal cord and brain *in vivo*, and in astrocytes derived from each region *in vitro*. Consistent with the accelerated appearance of astrocytes in the spinal cord relative to brain, we observed near parallel elevations in IL-6, including higher levels of expression in adulthood. Further supporting the regulatory link between IL-6 and GFAP, cultures of primary astrocytes derived from the spinal cord not only expressed greater GFAP, but also secreted more than 2-fold higher levels of IL-6 relative to parallel cultures of cortical astrocytes. Given the important role played by IL-6 in the response of the CNS to injury and disease, the current findings have potentially important implications for understanding differential responses across the neuraxis. What drives higher IL-6 expression in the spinal cord overall, and in isolated spinal cord astrocytes compared to those derived from cortex, remains to be established.

The transcription factor STAT3 is a key signaling partner for IL-6 and plays integral roles in CNS responses to injury. It is of potential significance therefore that we observe increased levels of STAT3 in the spinal cord relative to brain developmentally and in adulthood. For example, while STAT3 protein and RNA levels were uniform in brain homogenates over the postnatal period to adulthood, levels in the spinal cord showed progressive increases, paralleling increases in IL-6 and GFAP. STAT3 is solidly linked as a driver of astrogliosis after SCI [57], such that mice with conditional STAT3 deletion in astrocytes show reductions in certain aspects of reactive astrogliosis, including cell hypertrophy, up regulation of GFAP, and scar formation in experimental SCI [57, 58] and in experimental autoimmune encephalomyelitis (EAE) [59, 60]. Mice lacking STAT3 in astrocytes also show reduced astrogliosis in animal models of traumatic brain injury [61], and stroke [62]. The important role of astrogliosis in the resolution of many neuropathologies is highlighted by findings that the outcomes in SCI and EAE are exacerbated in mice lacking astrocyte STAT3, with increased spread of inflammation, infection, and impaired recovery of function [57, 58, 63–65]. Collectively these results suggest the need to further understand the significance of the coordinate elevations observed in GFAP, IL-6 and STAT3 in the developing and adult spinal cord compared to the brain and any regional differences occurring in response to injury and disease.

### GFAP reactivity is greater in the spinal cord compared to brain after focal white matter demyelinating lesions

GFAP is up regulated in the ageing brain, after CNS injury, and in many disease states [50]. Much remains to be learned regarding the specific roles GFAP plays in injury and repair responses. To begin to address whether the increases we observe in GFAP expression in the spinal cord relative to brain are also reflected in responses to injury, we compared changes in response to a focal demyelinating injury. Our initial focus on white matter responses relates to the fact that GFAP was first purified from an MS plaque [66] with astrocytes being widely observed in demyelinating conditions [67], including MS [68, 69], Alexander Disease [70], Megalencephalic leukoencephalopathy with subcortical cysts [71], and vanishing white matter disease [72]. As in other neurological conditions, there appears to be a dual role for astrocytes in demyelinating disease with both beneficial and detrimental attributes reported [69, 73–75]. Overall, the impact of astrocytes may depend both on the cause of injury, its location, and stage. It is interesting therefore that results in the current study show that GFAP increased to a higher level in the spinal cord white matter compared to the corpus callosum at the same time point (14 d) after a focal demyelinating injury. Interestingly, overall increases observed in ALDH1L1 in each region were very similar. Altogether these findings point to higher levels of GFAP per ALDH1L1 positive astrocyte in the spinal cord compared to the brain white matter in response to focal demyelination.

While the significance of the enhancements we observe in GFAP expression by astrocytes in spinal cord white matter compared to the corpus callosum after focal demyelination remain to be determined, we can gain some insight from prior studies demonstrating key roles for astrocytes and GFAP in myelin maintenance and repair. For example, studies of GFAP knockout mice suggest that GFAP is essential for white matter integrity with mice lacking GFAP exhibiting hypomyelination and a loosening of myelin wraps [46]. GFAP also interacts with GLAST and therefore may affect glutamate clearance [76]. Also, astrocyte–derived BDNF supports myelin protein synthesis after demyelination [77]. Astrocytes can form gap junctions with oligodendrocytes [78, 79] that may be crucial for maintenance of ionic balance and transmitting energy for oligodendrocyte function [80]. However, in other contexts astrocytes may accelerate myelin loss by playing a role in autoimmunity as antigen presenting cells, or by releasing chemokine’s that regulate T cell activity [81, 82]. Other studies have shown that astrocytes inhibit autoimmunity by expressing factors that block T cell differentiation [74], or trigger their apoptosis [83]. Reflecting the dual and likely stage-dependent actions of astrocytes, in a model of osmotic demyelination, astrocytes exerted detrimental effects early by secreting pro-inflammatory cytokines, but neuroprotective effects later by up regulating NGF and GDNF [84]. While much remains to be learned regarding the roles of astrocytes and GFAP in demyelination and myelin repair, the results presented here suggest that spinal cord astrocytes have the capacity to express more GFAP compared to those in brain, developmentally, in adulthood, and in response to focal demyelinating injury.

There are several key implications arising from the findings of the current study. First, these studies underscore regional astrocyte heterogeneity by highlighting their earlier appearance and higher levels of expression of GFAP, IL-6 and STAT3 in the spinal cord relative to brain. Since these findings extend to cell culture, the current studies point to the importance of utilizing astrocytes form either brain or spinal cord when attempting to model responses relevant to each region *in vitro*. Also, since spinal cord astrocytes express higher levels of GFAP in the context of demyelination relative to those in the corpus callosum, our findings provide additional support for the idea that astrocyte heterogeneity across the neuraxis is positioned to differentially impact the response of the brain and spinal cord to injury and disease, and that these differences will need to be taken into account in the design of targeted therapies.
